# Supplementary material for: Machine learning augmented diagnostic testing to identify sources of variability in test performance
Source: PLoS Comput Biol. 2025 Nov 4;21(11):e1013651. doi: 10.1371/journal.pcbi.1013651 (PMC12646444; doi:10.1371/journal.pcbi.1013651)
Supplement: S1 Text — Including model structure and fitted posteriors of TBMI-lite model parameters, for both Derbyshire and Devon areas. (PDF) [file pcbi.1013651.s001.pdf]

Machine learning augmented diagnostic testing to identify sources of variability in test performance
Supplementary Information

Christopher J. Banks[1], Aeron Sanchez[1], Vicki Stewart[2], Kate Bowen[2], Thomas Doherty[3], Oliver Tearne[4], Graham Smith[5], and Rowland R. Kao[1,6,*]

[1]Roslin Institute, University of Edinburgh, UK
[2]UK Farmcare Ltd., Stone, UK
[3]Department of Mathematics and Statistics, University of Strathclyde, UK
[4]The Animal and Plant Health Agency, Weybridge, Surrey, UK
[5]National Wildlife Management Centre, Animal and Plant Health Agency, Sand Hutton, York, UK.
[6]School of Physics and Astronomy, University of Edinburgh, UK
[*]Correspondence: rowland.kao@ed.ac.uk

October 24, 2025

Simulation model methods

Model construction

The fundamentals of the modeling approach are similar to a previously published model for national BTB transmission [1] but here, considering only a more limited regional level of disease transmission under regular annual testing.

We allow for the following discrete individual animal states. All cattle are born susceptible (**S**), when infected enter a non-infectious but test sensitive state (**T**), and then proceeding on at a fixed rate to an infectious stage (**I**). The sensitivity of the test is fitted separately for each of the two infected states. In addition, infectious stage cattle are assumed to seed the local environment, allowing for continued transmission after cattle are moved away or slaughtered—this environmental reservoir incorporates the role of badgers.

Infection between cattle and badgers is assumed to be uniform within geographically defined hexagonal tiles, with each tile having single badger density estimate (i.e. uniform in each tile). Dynamics of infection in badgers is explicitly included in the model fit at the badger social group (or "group") level. The local density of groups is imputed based on estimates of badger main sett densities at a 500m $\times$ 500m square grid resolution, previously calculated by Croft et al. [2], but with revised, more recent density estimates. Assuming a main sett can be associated with a single group, hereafter imputed badger group densities at the grid cell level are referred to as IBDs.

Badger densities in England and Wales are known to be increasing, particularly in types of land that predominate in high incidence areas of England and Wales but are less common elsewhere [3]. Therefore as part of the parameter inference we fit an annual increase in IB infection pressure in annual and six monthly testing areas in England and Wales (only), using 2013 as the baseline—the end of the last national badger survey (NBS) on which our IBD's are based.

SICCT testing

Approximately 90% of all tests recorded in the SAM database are implemented in the simulation, as stated in a recent APHA surveillance report [4]. We omit radial and contiguous tests and gamma interferon testing. Area tests, as implemented since 2013 in low risk areas, are also not relevant to the regions in our simulations. We only consider a single interpretation of the SICCT test, i.e. we do not distinguish between the standard interpretation used for the majority of testing, and the severe interpretation as used in herds where OTF status is lost. We include pre- and post-movement testing, triggered for the appropriate movements of cattle as required by policy, incorporating all changes since first introduced in 2006. Pre- and post-movement tests are otherwise treated as with routine SICCT testing, including triggering a whole herd test at the source herd. Positive reactors from routine skin tests are triggered based on the current test sensitivities (parameters iii and iv, below). All reactors are culled and removed from the model. Testing events are assumed to be independent of each other, i.e. a prior false negative has no influence on the likelihood of a false negative at a future test.

A positive reactor from any SICCT test or routine lesion test will result in a location entering breakdown status and being OTFS/OTFW. All animals in a breakdown location that are in OTFS/OTFW undergo a movement restriction

until the location exits breakdown. A confirmation lesion test is performed on the reactor and if confirmed, the location enters OTFW. Otherwise it remains OTFS. If the premises is in OTFW then it must pass (without any reactors) two short interval tests (SITs) to exit this OTFW status, at which point movement restrictions are lifted. It must then pass a 6 month and then 12 month follow up test to return to routine surveillance tests. If any of the SITs fail then it has to pass two SITs again. If any of the follow up tests fail it re-enters OTFS/OTFW status and must follow through the short interval and follow up test flow again. If the location remains OTFS then it must only pass one SIT to exit OTFS status and start follow up tests. Current regulations, where OTFS herds must pass two SITs in most cases, are not yet implemented. For locations under annual testing, they must go also through 6 and 12 month follow up tests.

Cattle movements

Cattle are moved between premises using data from the cattle tracing system (CTS). The recorded pattern of movements in part reflects changes in TB infection status, however, this creates possible inconsistencies in simulation. Here, when a premises' OTF status is removed (either withdrawn, OTFW, or suspended, OTFS, both of these resulting in a breakdown), recorded CTS movements from those premises should not occur – they do in reality because infection never occurred but in simulation, identification of an infected animal require that they be stopped. We therefore, in simulation, suspend all recorded movements until after OTF status is reacquired, at which point these movements are released immediately. The total volume of movements and livestock are retained on a holding-by-holding basis, but the timing of movements is changed. If many movements are released at once this may result in a pulse of infectious movements (should the removal of restriction leave some infected cattle) however in the fitted simulations, investigations show that the number of infected cattle involved in these movements are few. Movements to market are ignored as a natural consequence of the 15-day increment used in the model (see below). It is also likely to be rare that livestock remain at market more than 6 days and therefore infections occurring at market likely to be negligible. Cattle with "death movements" and cattle moving to premises out of the simulated region are removed from the model. Movements to slaughterhouse (either within or out of the region) trigger a routine lesion test. Movements trigger pre- and post-movement tests, as required. Cattle moving into the simulated region are assumed to be uninfected, and movements of infected cattle out of the simulated region are therefore considered to be lost (i.e. they do not contribute further to infection in the region, even by indirect effects).

Parameter inference

Parameter inference is conducted via an Approximate Bayesian Computation (ABC) scheme with sequential Monte Carlo (SMC) sampling, following previous related work [1, 5]. An important consideration is the choice of observations used to calibrate the models. In principle, the entire testing record could be used, however this computational challenge lies beyond the scope of the current project. We fit the observed number of failed herd level tests and reactors but use regularised hexagonal cells as the unit of fit. Individual cattle are tracked as they move (in the recorded CTS data) from premises-to-premises, allowing for the tracking of individual life histories. We combine two summary statistics that capture multiple features in the data: (a) number of reactors per region per year and (b) number of failed tests (i.e. number of times there is at least one confirmed reactor when a herd is tested) per region per year.

In each generation of the ABC-SMC scheme, a particle is generated with a single set of values for the fitted parameters, sampling from the model priors in the initial round of the ABC-SMC scheme, and in following rounds sampling from the particles with weights generated during the previous round. The particle is then perturbed using a Gaussian kernel. A single generation of the scheme is completed after 1,000 accepted particles are generated. We generate 1,000 accepted parameter sets for each round of ABC based on the threshold defined from the distribution of sample statistics from the previous round. Acceptance is based on a comparison of the following metric to the defined threshold:

$$score = \sum_{cells} \Big(\, (ft_{sim} - ft_{obs}) * \max{(r_{obs})} \Big)^2 + \sum_{cells} \big((r_{sim} - r_{obs}) * \max{(ft_{obs})} \big)^2$$

where: $score$ is a single particle score, ft_{sim}/ft_{obs} are numbers of failed tests per county simulated/observed, r_{sim}/r_{obs} is the number of reactors per county simulated/observed.

Model runtime optimisations

Model simulations increment in 15 day time steps, aggregating recorded data at this temporal resolution. Where this approach creates an anomaly in the movement pattern (e.g. has multiple moves in the same timeframe) we retain only the location where cattle have been resident longest in that timestep. Infectious cattle that pass through some short term locations will not apply infection pressure at these locations, because these locations have been stripped out.

The individual livestock disease infection status is updated probabilistically at the same 15 day intervals, where there are two types of events: infection caused by either density dependent cattle-to-cattle transmission or transmission from infected badger groups in the same grid cell, or infection state transition rates. Similarly, in a grid cell, badger social groups are infected by infectious cattle, or by other infected groups.

The simulation steps through time in fixed 15 day increments. Only integer increments are allowed in a year, resulting in the first and last periods being two days longer.

Model initialisation

For each simulation run, each individual animal on record at the beginning of the data period (01/01/2006) is given a starting locations and disease state, within locations of infected cattle chosen as outlined in parameter xi (below). No infected badger groups are included at initialisation. Under this specification, the influence of the initial conditions appear to be negligible after a burn-in period of five years. The model is run to 31/12/2020 i.e. a period of 15 years, including burn-in.

Routine skin tests dates are uniformly allocated across the testing period, i.e for annual testing areas, uniformly in the first 12 months, and equivalent for six monthly and four yearly testing areas. Further tests are scheduled assuming a uniform distribution across the specified testing interval. There is no seasonal variation in testing rate considered.

For each simulation step the following tasks are executed:

1. Individual animal CTS movements are processed.
2. Using the current estimate of the parameters, disease transmission events per susceptible animal are processed. Test Sensitive to Infectious transition events are also processed at rate γ. See Supplementary Material Section 1 in Brooks-Pollock et al. [1] for reference.
3. Scheduled, routine skin tests are processed with whole herd tests for annual and six-monthly, and part herd for four yearly testing. Sensitivity taken from parameters. If any breakdowns are detected, further skin or confirmation lesion tests are scheduled, based on the OTFS/OTFW testing regime flow. If there are no reactors a new routine test is scheduled based on historically accurate parish testing interval (PTI) values. The code is formulated to allow for quarterly changes at the parish level. The PTI values vary from 6 months to 4 years and depend on the historically defined risk areas and period specific policies.

Model parameters and initial conditions

The model includes 11 fitted parameters:

i. Susceptible $\rightarrow$ Test Sensitive rate for cattle (β)

ii. Test sensitive $\rightarrow$ Infectious rate for cattle (γ)

iii. Infectious animal SICCT test sensitivity (standard interpretation)

iv. Test-sensitive animal SICCT test sensitivity (standard interpretation)

v. Routine Lesion test sensitivity

vi. Confirmation Lesion test sensitivity

vii. Rate at which infectious cattle infect local (around premises) badger groups (β_{CB}).

viii. Rate at which infected badger groups infect other badger groups within a local area (here defined as a local hexagonal grid cell) (β_{BB}).

ix. Rate at which infected badger groups infected local cattle (β_{BC}).

x. Rate at which infected badger groups become susceptible again. (μ).

xi. Seeding:

(A) A proposal for the number of starting infections or seeds is generated from the prior (in the first generation) or the previous generation posterior.

(B) For each hex cell, a farm is randomly chosen from the list of possible CPHs and based on the size of farm and the load factor for that region the number of seeds to be sampled for that farm is calculated.

(C) Using sampling without replacement, the number of animals calculated in (B) is sampled at that farm and are marked as seeded infected animals.

(D) The steps in (B) and (C) are repeated until the required number of seeds for that region are met.

(E) With a full list of seeding animals now gathered, they are all given an infectious state for the first time step.

Fitted Parameter	Derbyshire Value	Devon Value
badgerDecay	0.285 [0.065-0.483]	0.275 [0.0637-0.472]
badgerToBadger	0.00465 [0.00081-0.00982]	0.00653 [0.00083-0.0147]
badgerToBadgerLocalMult	0.0502 [0.00654-0.0948]	0.0437 [0.00774-0.0875]
badgerToCattle	0.00105 [0.000096 - 0.00275]	0.00146 [0.000111-0.00342]
cattleToBadger	0.0843 [0.00527-0.185]	0.0758 [0.00836-0.166]
eToIRate	8.8e-05 [4.77e-05 - 1.42e-06]	5.35e-05 [3.23e-05 - 8.45e-05]
sToERate	0.0276 [0.011-0.0440]	0.0208 [0.0079-0.0389]
falsePositiveRate	0.000260 [0.000071-0.000418]	5.55e-05 [9.19e-06 - 1.09e-4]
infectiousSensitivity	0.751 [0.508-0.937]	0.704 [0.405-0.927]
numSeeds	3520 [1720-4850]	1160 [3810-1920]

Table 1: Fitted posteriors of TBMI-lite model parameters, for both Derbyshire and Devon areas.

Following previous analyses [1], we fit only the SICCT individual test sensitivity (i.e. test specificity is assumed to be very high) and we do not consider independently the SICCT standard and severe interpretations (i.e. the distinction in test interpretations used that are dependent on herd OTF status). The individual test sensitivity should therefore be interpreted as the net sensitivity that best captures the metric in the model. We note that this is different from the HSp and HSe, as used in the diagnostics evaluation in the main text.

As well as the fitted parameters above, the following parameters (with imposed or calculated values) are relevant to the badger model:

xii. N_b^i is the total number of **badger groups** in cell i

xiii. N_c^k is the total number of **cattle** at premises k

xiv. I_b^i is the number of **infectious badger groups** in cell i

xv. I_c^k is the number of **infectious cattle** at premises k

xvi. $p_c^i := \frac{\sum_{k\in i} I_c^k}{\sum_{k\in i} N_c^k}$ is the **proportion of infectious cattle** in hex cell i

Assuming density dependence, the equation governing the number of infected setts in cell i is

$$\frac{dI_b^i}{dt} = \left(\beta_{cb} p_c^i + \beta_{bb} I_b^i\right)\left(N_b^i - I_b^i\right) - \mu I_b^i$$

This implies that in a local cell with badgers but no cattle, a basic reproduction number of ${R_0}^{bb} = \frac{\beta_{bb} N_b^i}{\mu}$. When less than one this would imply badger infection cannot persist on its own. If there are no infectious cattle but ${R_0}^{bb} > 1$, the infection in badger groups would reach an equilibrium state, where on average the number of infected groups in a grid cells i is $I_b^i = N_b^i - \frac{\mu}{\beta_{bb}}$.

Posterior parameter estimates

The fitted parameter posteriors are detailed in Table 1.

License

For the purpose of open access, the author has applied a Creative Commons Attribution (CC BY) licence to any Author Accepted Manuscript version arising from this submission.

References

[1] Brooks-Pollock E., Roberts G.O., and Keeling M.J. (2014). "A dynamic model of bovine tuberculosis spread and control in Great Britain". *Nature*, 511(7508):pp. 228–231. ISSN 1476-4687. URL http://dx.doi.org/10.1038/nature13529.

[2] Croft S., Chauvenet A.L.M., and Smith G.C. (2017). "A systematic approach to estimate the distribution and total abundance of British mammals". *PLOS ONE*, 12(6):p. e0176339. ISSN 1932-6203. URL http://dx.doi.org/10.1371/journal.pone.0176339.

[3] Judge J., et al. (2014). "Density and abundance of badger social groups in England and Wales in 2011–2013". *Scientific Reports*, 4(1):p. 3809. ISSN 2045-2322. URL http://dx.doi.org/10.1038/srep03809.

[4] Animal and Plant Health Agency (2020). "Bovine TB epidemiology and surveillance in Great Britain, 2019". https://www.gov.uk/government/publications/bovine-tb-epidemiology-and-surveillance-in-great-britain-2019.

[5] Orton R.J., et al. (2018). "Identifying genotype specific elevated-risk areas and associated herd risk factors for bovine tuberculosis spread in British cattle". *Epidemics*, 24:pp. 34–42. ISSN 1755-4365. URL http://dx.doi.org/10.1016/j.epidem.2018.02.004.
